# A multilayered approach to the analysis of genetic data from individuals with suspected albinism

**DOI:** 10.1136/jmg-2022-109088

**Published:** 2023-07-17

**Authors:** Panagiotis I. Sergouniotis, Vincent Michaud, Eulalie Lasseaux, Christopher Campbell, Claudio Plaisant, Sophie Javerzat, Ewan Birney, Simon C. Ramsden, Graeme C. Black, Benoit Arveiler

**Affiliations:** 1 Division of Evolution, Infection and Genomics, School of Biological Sciences, Faculty of Biology, Medicine and Health, University of Manchester, Manchester, UK; 2 European Molecular Biology Laboratory, European Bioinformatics Institute (EMBL- EBI), Wellcome Genome Campus, Cambridge, UK; 3 Manchester Centre for Genomic Medicine, Saint Mary’s Hospital, Manchester University NHS Foundation Trust, Manchester, UK; 4 Manchester Royal Eye Hospital, Manchester University NHS Foundation Trust, Manchester, UK; 5 Department of Medical Genetics, University Hospital of Bordeaux, Bordeaux, France; 6 INSERM U1211, Rare Diseases, Genetics and Metabolism, University of Bordeaux, Bordeaux, France

**Keywords:** Genetics, Medical, Genetic Testing, Ophthalmology

## Abstract

Albinism is a clinically and genetically heterogeneous group of conditions characterised by visual abnormalities and variable degrees of hypopigmentation. Multiple studies have demonstrated the clinical utility of genetic investigations in individuals with suspected albinism. Despite this, the variation in the provision of genetic testing for albinism remains significant. One key issue is the lack of a standardised approach to the analysis of genomic data from affected individuals. For example, there is variation in how different clinical genetic laboratories approach genotypes that involve incompletely penetrant alleles, including the common, ‘hypomorphic’ *TYR* c.1205G>A (p.Arg402Gln) [rs1126809] variant. Here, we discuss the value of genetic testing as a frontline diagnostic tool in individuals with features of albinism and propose a practice pattern for the analysis of genomic data from affected families.

## Background

Albinism is a group of conditions associated with reduced levels of melanin pigment that result in developmental visual system anomalies; skin and hair manifestations are also present in the majority of cases. Significant phenotypic variability has been described among affected individuals. The pigmentation of the skin, the hair and the uveal layer of the eye, for example, can range from very reduced to (almost) normal. The ophthalmic features, which are the hallmark of the condition, are also variable and can include nystagmus, reduced best-corrected visual acuity, photoaversion, iris transillumination, foveal hypoplasia and chiasmal misrouting of the optic nerves.^1^

At least 20 genetically distinct forms of albinism have been described. These can be caused by variants in genes encoding: melanogenic enzymes (*TYR*, *TYRP1* and *DCT*); channels regulating melanosomal pH (*OCA2* and *SLC45A2*) or calcium homeostasis within melanin-containing cells (*SLC24A5*); subunits of multiprotein complexes that are involved in melanosome maturation and/or trafficking of melanogenic enzymes (Hermansky-Pudlak syndrome [HPS] group of genes).^2^ Most forms of albinism are inherited as autosomal recessive traits apart from *GPR143*-related albinism, which is an X-linked recessive condition. Although it is possible that additional albinism-associated genes remain to be discovered, alterations in such genes are expected to explain only a very small fraction of cases. Indeed, pathogenic variants in two genes that have been recently implicated in albinism, *DCT* and *BLOC1S5*, were detected in only 2 of 1500 (0.13%) albinism cases each.^3 4^

Obtaining a precise genetic diagnosis in people with albinism:

- Allows timely identification of the subset of affected individuals who are at risk of developing major complications (lung fibrosis, prolonged bleeding and/or granulomatous colitis in individuals with certain subtypes of HPS; immunodeficiency and neurodegeneration in individuals with Chediak-Higashi syndrome [CHS]). In these cases, early diagnosis can drive evidence-based changes in care management and result in improved outcomes (a relevant example can be found in the Cases section: proband 1).
- Helps rule out differential diagnoses in individuals presenting with non-specific features such as nystagmus and/or foveal hypoplasia.
- Facilitates accurate genetic counselling and can inform reproductive planning in affected individuals and families.

To reach a genetic diagnosis, integration of phenotypic and genomic information is required; family context and dynamic changes in pigmentation should also be taken into account. In this article, we discuss clinical genetic testing in individuals who have features of albinism and propose a molecular diagnostic decision tree.

## Approach to genetic testing in albinism

An initial aim of genetic testing in individuals suspected of having albinism is the identification of single-nucleotide variants (SNVs) and copy number variants (CNVs) impacting albinism-related genes. Additional analysis of other genes — such as *FRDM7*, *SLC38A8* and *PAX6* — is recommended; although these genes have been implicated in distinct conditions that do not generally feature hypopigmentation, the associated phenotypic spectra include characteristic ophthalmological manifestations of albinism. Notably, infants whose presentation is linked to changes in these three genes may have lightly pigmented skin/hair due to their familial/genomic background; this can increase the apparent overlap between these conditions and albinism.^5^

High-throughput DNA sequencing approaches are typically used for genetic variant detection. These approaches can be complemented by high-resolution array CGH (comparative genomic hybridization) analyses that focus on CNVs, which are thought to represent >10% of pathogenic alleles.^5^ It is noted that bioinformatic tools that mine sequencing data are increasingly replacing array CGH-based workflows.

**Figure 1:**
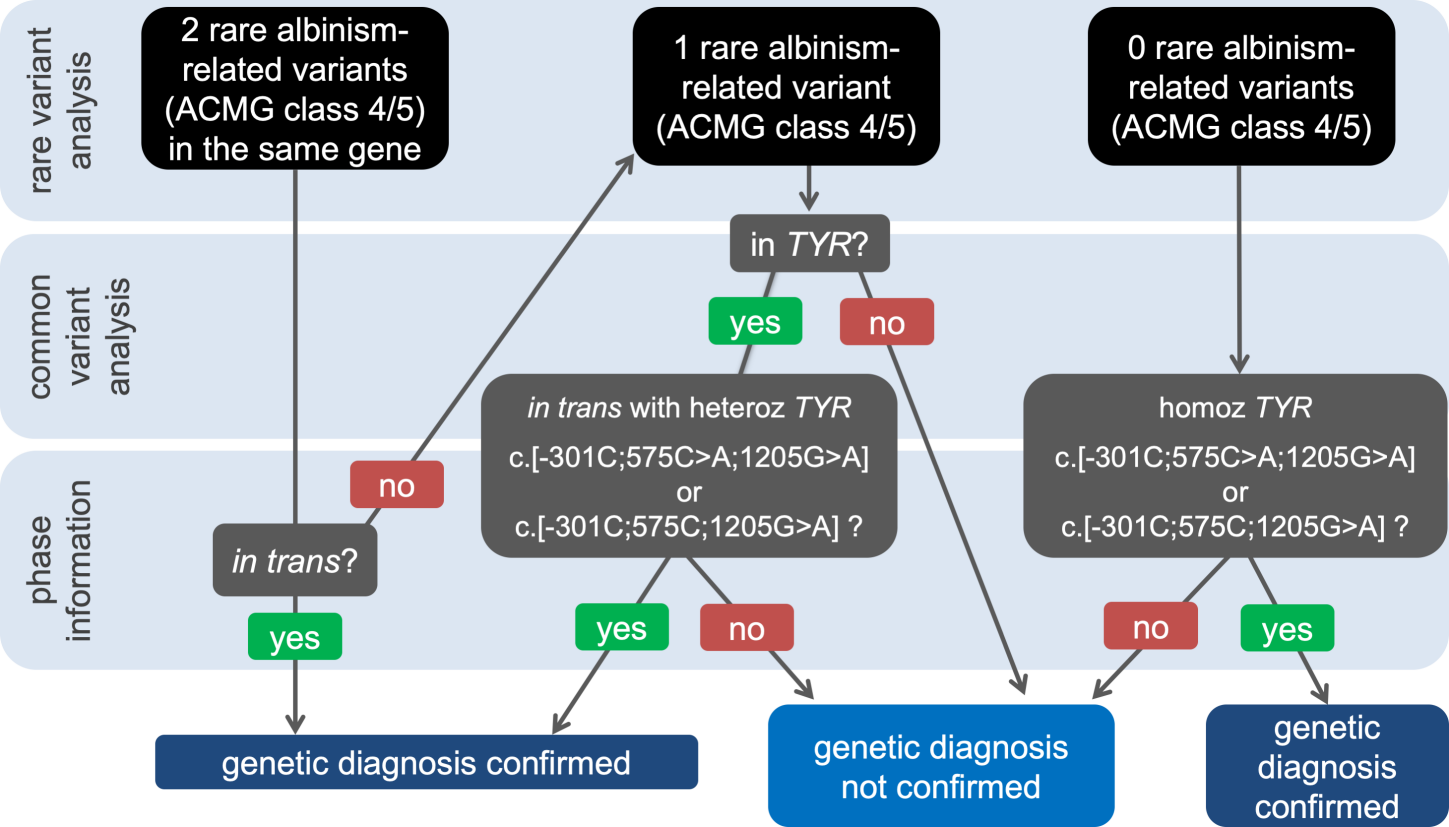
Schematic showing the proposed workflow for downstream analysis of genetic variants detected in individuals who have features of albinism.The focus of this decision tree is diagnostic genetic testing. Predictive testing, either in the context of a specific family or in broader settings requires a customised approach. Notably, as genotypes that include albinism-associated *TYR* haplotypes are unlikely to be fully penetrant, accurate risk prediction remains challenging.For brevity, this graph focuses on autosomal recessive forms of albinism and the X-linked, *GPR143*-related subtype is omitted.ACMG, American College of Medical Genetics and Genomics; heteroz, heterozygous; homoz, homozygous.

Like other rare genetic disorders, molecular diagnostic practice patterns for albinism rely heavily on a search for low-frequency (rare), highly penetrant genetic variants. This reflects the wide acceptance of the rare disease – rare variant hypothesis which states that, if a disorder with a significant genetic component is rare, then the underlying genetic defect(s) will also be rare. However, in the past decade, a number of studies have challenged the universal nature of this paradigm and have highlighted the role of common genetic variation in rare phenotypes.^6 7^ In light of this and the findings of our recent work on albinism,^8^ we propose a three-step approach to the analysis of genetic data from patients with suspected albinism, *i.e.* individuals with at least one of the key ophthalmic features of the condition (for example prominent foveal hypoplasia) (figure 1).

### Rare variant analysis

It is recommended that the initial focus remains on the detection and classification of rare SNVs and CNVs in relevant genes. Established criteria should be used to analyse these changes. Presently, most genetic laboratories use the variant classification system developed by the ACMG-AMP (American College of Medical Genetics and Genomics and the Association for Molecular Pathology).^9 10^ This involves scoring genetic alterations and assigning them into one of the following five classes: benign (class 1), likely benign (class 2), variant of uncertain significance (VUS) (class 3), likely pathogenic (class 4) and pathogenic (class 5).

The presence of two class 4 or 5 variants in a gene of interest confirms a genetic diagnosis after phase has been established (unless the relevant gene is on the X-chromosome or it is associated with an autosomal dominant inheritance pattern, in which case, one such change can be sufficient). Variants of uncertain significance cannot contribute to a high-confidence genetic diagnosis that guides clinical decision making but are worth reporting, sharing in open-access databases (such as ClinVar) and being considered for reclassification at a later stage.

### Common variant analysis

Common variation in the *TYR* gene (encoding tyrosinase) has been implicated in relatively mild forms of albinism since the early 1990s.^11–13^ We have recently shown that the presence of haplotypes that include specific alleles of three common *TYR* variants has similar effects to rare sequence alterations that have high penetrance.^8^ These three common variants are

- *TYR* c.-301C>T [rs4547091], located in the promoter, with an allele frequency of ~60% (for the effect allele which can be described as c.-301C or c.-301) in non-Finnish European (NFE) populations in the Genome Aggregation Database (gnomAD v2.1.1).
- *TYR* c.575C>A (p.Ser192Tyr) [rs1042602], with a minor allele frequency of ~36% in gnomAD NFE populations.
- *TYR* c.1205G>A (p.Arg402Gln) [rs1126809], with a minor allele frequency of ~27% in gnomAD NFE populations.

The relevant albinism-associated haplotypes are

- *TYR* c.[−301C;575C>A;1205G>A]; this can also be described as *TYR* c.[−301=;575C>A;1205G>A] or [C;A;A]; the frequency of this haplotype in people with European-like ancestries is ~1%.
- *TYR* c.[−301C;575C;1205G>A]; this can also be described as *TYR* c.[−301=;575=;1205G>A] or [C;C;A]; the frequency of this haplotype in people with European-like ancestries is ~0.3%.

It is recommended to seek these two rare haplotypes in the genetic data of individuals with suspected albinism who carry the *TYR* c.1205G>A change. Current evidence suggests that the presence of these haplotypes, either in the homozygous state or *in trans* to a *TYR* class 4 or 5 variant, would be sufficient to establish the genetic diagnosis, especially in people who have relatively mild forms of albinism (a relevant example can be found in the Cases section: proband 2). It is highlighted that the *TYR* c.-301C and the *TYR* c.575A (p.Ser192Tyr) alleles are frequently found in linkage disequilibrium so that the presence of the latter would, in most cases, suggest the presence of the former on the same haplotype.

### Obtaining phase information

At present, the data generated by most DNA sequencing workflows take the form of unphased genotypes. As a result, it is generally not possible to directly observe on which of the two parental chromosomes (or haplotypes) a particular allele falls on. To address this, haplotype phasing must be performed in order to assign genetic variants to the corresponding paternal or maternal chromosomes.

Typically, the focus of haplotype phasing is heterozygous genotypes. Two main phasing methods have been described: mendelian phasing (which uses parental genotypes) and statistical phasing (which uses intermarker correlation). More recently, advances in long-read sequencing technologies have enabled phasing without relying on statistical inference or testing relatives.^14^ Nonetheless, the most commonly used phasing method in clinical practice at present involves analysing parental samples. This approach is imperfect as obtaining appropriate samples from relatives can be challenging; the analysis may uncover misattributed genetic relationships; testing of multiple individuals over more than one generation may be required to obtain fully informative genotypes.

In the context of albinism, phase information can help to (i) identify compound heterozygosity (for example, when two rare variants are detected) or (ii) to clarify the haplotypic structure around the *TYR* locus when common albinism-associated changes are present (a relevant example can be found in the Cases section: proband 3). In these scenaria, phasing can be key to obtaining a high-confidence genetic diagnosis.

## Cases

Three cases are discussed further to illustrate the utility of genetic testing in albinism (proband 1) and to highlight the value of the proposed molecular diagnostic approach (probands 2 and 3).

Proband 1 was diagnosed with oculocutaneous albinism in the first few months of life. He presented with nystagmus and was found to have iris transillumination, fundal hypopigmentation, and a featureless fovea. His skin and hair were moderately pigmented and there were no other concerns about his health/development. There was no family history of albinism or visual problems. Genetic testing was requested and revealed a homozygous *HPS5* c.1507G>T (p.Glu503Ter) variant. Pathogenic variants in *HPS5* are known to cause a form of HPS that is characterised by albinism and dysfunction of blood platelets (leading to prolonged bleeding). The infant was referred to the paediatric haematology department where he had platelet function analysis. This revealed prolonged PFA-100 closure time following stimulation with collagen and epinephrine (>300 s, reference range 79–205 s). This finding had significant implications as the child was due to have a routine urological procedure, which was safely performed with appropriate precautions. Notably, in our cohorts, approximately 1 in 20 individuals with suspected albinism is found to have biallelic pathogenic alterations in HPS-related or CHS-related genes.^5^ This case is also discussed in a previous report by our group where the benefits of genetic testing over more invasive and labour-intensive haematological approaches for the diagnosis of HPS are highlighted.^15^

Proband 2 was noted to have nystagmus soon after birth. There were no other medical concerns and there was no family history of relevance. The diagnosis of infantile nystagmus was made and the proband was followed up in his local ophthalmology unit for a number of years. An assessment in a tertiary ophthalmic genetic clinic was initiated in middle childhood, and the vision at that point was 0.3 LogMAR (Logarithm of the Minimum Angle of Resolution) in each eye. His irides were blue and there were no transillumination defects. Optical Coherence Tomography (OCT) imaging revealed foveal hypoplasia. It is noted that the broband’s hair were light brown and his skin colour was similar to that of close relatives. Genetic testing was initiated, revealing homozygosity for the *TYR* c.[−301C;575C>A;1205G>A] or (C;A;A) haplotype described previously. This genotype has been shown to confer a high ‘risk of albinism’ and should be considered pathogenic/predisposing.^8^

**Figure 2:**
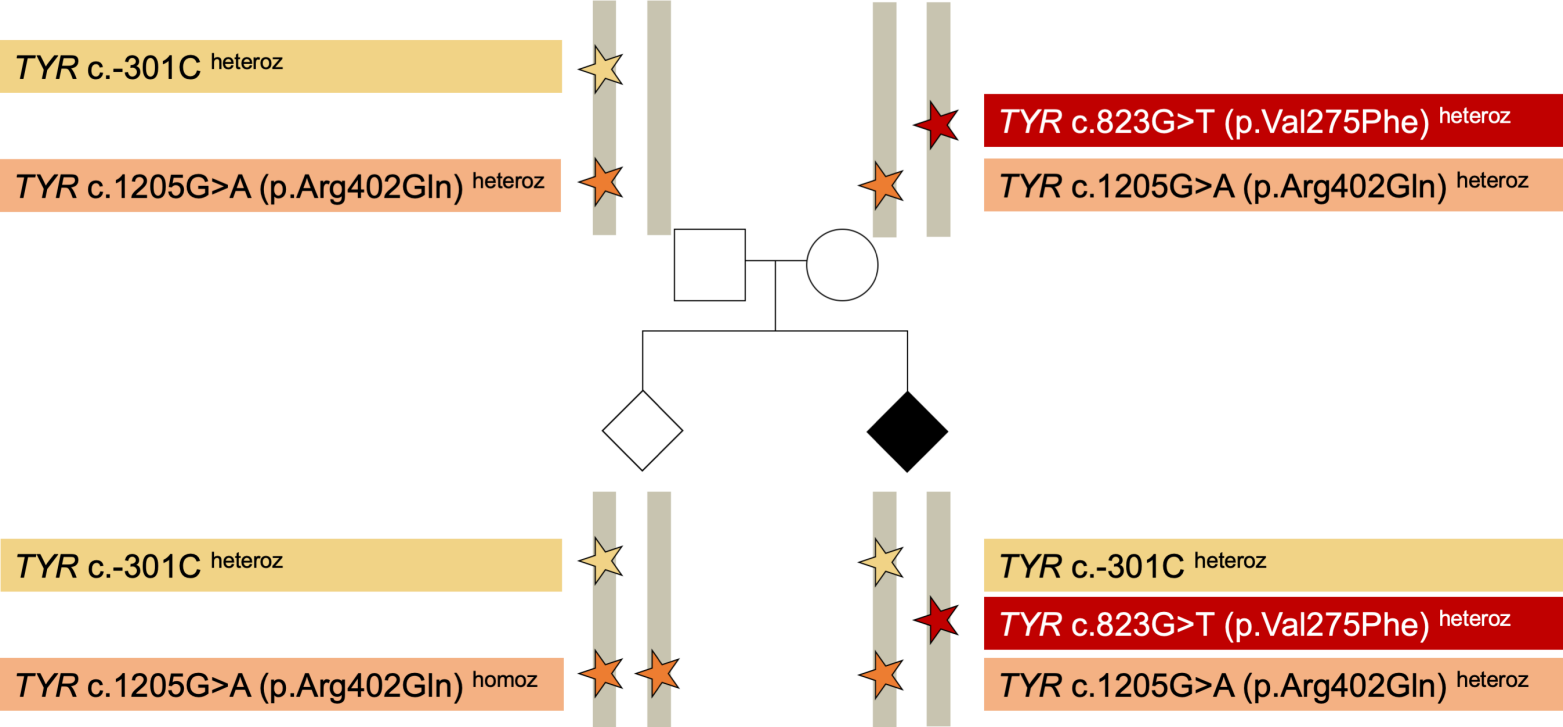
Example of how the analysis of a common *TYR* promoter variant can help obtain a genetic diagnosis in people with albinism (see also Cases section: proband 3). It is highlighted that the *TYR* c.-301C allele is predicted to reduce *TYR* gene expression in fetal retinal pigment epithelia by altering an *OTX2* binding site in the *TYR* promoter.^16 17^ In contrast, the *TYR* c.1205G>A missense change produces a temperature-sensitive enzyme that has reduced catalytic activity and a tendency to be retained in the endoplasmic reticulum.^11 17–20^ Notably, the *TYR* c.-301 position is unlikely to be targeted by many currently used genomic assays (including certain panel-based and exome sequencing tests), and expansion of the target enrichment design to include this region may be required.heteroz, heterozygous; homoz, homozygous.

Proband 3 has a diagnosis of oculocutaneous albinism. Conventional genetic analysis focusing on rare variants revealed a heterozygous likely pathogenic variant in *TYR* c.823G>T (p.Val275Phe) [rs104894314]; this was found to be inherited from the proband’s mother (figure 2). When the common, functionally-relevant *TYR* c.1205G>A (p.Arg402Gln) variant was included in the analysis, the proband appeared to have a genotype similar to that of his unaffected mother. Analysis of the common promoter variant *TYR* c.-301C>T, as well as of *TYR* c.575C>A (p.Ser192Tyr), and testing of samples from other family members highlighted that the proband carried the likely pathogenic *TYR* c.823G>T change *in trans* to the c.[−301C;575C;1205G>A] haplotype (figure 2). This rare haplotype is likely to be pathogenic/predisposing,^8^ and the fact that it is present *in trans* to a likely pathogenic change confirms the genetic diagnosis of albinism.

## Concluding remarks

We describe a framework for analysing genetic test results from individuals suspected of having albinism. The importance of incorporating selected common genetic variants in the analysis and the key role of haplotype phasing are highlighted.

Our work in particular clarifies the long-lasting debate about the impact of the *TYR* c.1205G>A (p.Arg402Gln) variant and its definition as a benign or pathogenic change.^11–13^ We argue that this variant can be considered to be pathogenic/predisposing in the context of specific haplotypes/genotypes, but not on its own.

In our setting, incorporating the two complex albinism-associated *TYR* haplotypes ([C;A;A] and [C;C;A]) in the analysis increased the diagnostic yield of genetic testing by 19%. This highlights the significant contribution of these changes to the genetic architecture of albinism.

It can be speculated that *TYR* c.1205G>A (p.Arg402Gln) is not the only missense *TYR* change whose effect can be modified by other variants. We expect that, in the future, genetic investigations for albinism will analyse key non-coding regions (including promoters and *cis*-regulatory elements) for all relevant genes. Notably, the concepts discussed here are likely to be relevant to the study of other rare disorders.
